# Our Summer at Maplewood. Chap. III. Still Dabbling in Dough

**Published:** 1875-04

**Authors:** 


### CHAPTER III.

### STILL DABBLING IN DOUGH.

"To-day, Alice, we will make rolls, French twist, and Graham bread. Let us begin with Graham bread. Having set the ferment last night, we now divide it, pouring a portion into a separate bowl, into which we stir Graham flour, adding more or less sugar, according to taste, and a little salt. Add flour, in small quantities at a time, and allow the sponge to rise during the intervals. When too stiff to stir readily, work well with the hand until the dough seems very elastic and clinging. Add, at this working, all the flour required. When stiff enough, the dough will work free from the sides of the bowl; but Graham bread should be made less stiff than white bread. Leave it in the bowl to rise; and, when light, knead it upon the board only enough to shape into loaves, and then put it in pans to rise.

"It is well to gash all loaves of bread with a sharp knife, after they are in the pan. This incision should be an inch deep, and extend nearly the length of the loaf. This prevents binding, and allows the loaf, in rising, more freedom to shape itself gracefully. Put it into warm pans, well covered, in a warm room. These Graham loaves will rise in twenty or thirty minutes, possibly fifteen. Graham bread does not require quite so hot an oven as white bread.

"For rolls and twist, we have a quart or three pints of ferment, into which we stir flour, the same as for bread. In every case be careful to have the flour warm, so that the fermentation may not be checked. Should you at any time, find that the ferment or sponge has received a chill, and is slow in its action, add the necessary warmth, not by placing it over a hot range, or close to the stove, where the heat will be extreme at some points, but put the bowl containing the sponge, in a pan of warm water, and stir it often until the whole mass of dough becomes of an even temperature. When this roll-sponge is thick enough to be worked by the hand, add to it an ounce of butter for each pint of ferment. If the butter is not perfectly sweet, use pure, fresh lard, instead. If you desire to have rolls light and spongy, work them at least half an hour in the bowl, adding flour very gradually. Then place the dough upon the molding-board and knead it well, adding flour only as is necessary to prevent it from sticking to the board and hands. The appearance of blisters upon the dough is an indication that it is well worked. Having washed and warmed the bowl and greased it with sweet lard or butter, return the dough to it, and let it rise. When light, loosen the dough from the edge of the bowl and it will come out clean. If the greasing is omitted, the dough adheres, and must be scraped from the bowl and then rubbed from the fingers by means of flour. This covers the molding-board with little hard wads, unfit to be worked into bread at this stage.

"Bring the dough quickly, with a few pressures, into a compact roll, and lay it aside. Cut from it a piece the size of a small loaf, and divide this into eight or ten parts, mold each in a round shape; and then, by rolling quickly back and forth under your hands, bring it into a long roll-form. Give the ends an extra roll, and you have pointed them slightly. Place this roll at one end of the bread-pan, and proceed in like manner to fill the pan with rolls. It is well to flour the sides of the rolls where they touch each other, as that will keep them from running together; and, when baked, they are easily separated.

"For the twist, take a piece of dough the size of a small loaf, and bring it into the long-loaf shape, pointing it at the end like the rolls; then, with a rolling-pin, flatten it like a pie-crust, rolling back and forth lengthwise, as it must be much longer than it is wide. Leave it an inch in thickness, and, with a sharp knife, cut it lengthwise into three strips of equal width. Leave these strips unseparated at the end farthest from you, and then carefully plait or braid them, pinching the ends well together. Lift the twist with care, and place it in a pan large enough to hold it without crushing or marring its form. If the dough is not allowed to get cold and is properly covered, these twists and rolls will rise in fifteen or twenty minutes; but remember, they should be lighter than bread. If not baked too brown, they will heat over nicely, by being placed for a few minutes in a hot oven and allowed to heat thoroughly, to the extent of taking on a brown tinge. A roll reheated I think better than one newly baked; but a roll dried for half an hour in a warm oven is another thing. Many cooks bake rolls too lightly; all bread is more digestible well baked.

"After testing them the same as bread, if you are still in doubt whether they are done, separate one from its fellow and press the finger against the soft part. If it retain the impression of the finger, it is not done. When baked sufficiently, it will rebound as soon as the pressure is withdrawn.

"When you wish to astonish your friends with a marvel of delicate lightness in rolls, add to the dough when worked nearly enough to go on the molding-board, the whites of three or four eggs beaten to a stiff froth, and an additional half ounce of butter for each egg. The butter should be worked in first; the eggs later and a little at a time, alternating with flour. If eaten the same day they are baked, these rolls are more delicate, being lighter, more spongy, and whiter, than without the egg; but they are also richer, and do not keep so well. In making these egg-rolls, work them almost entirely in the bowl, for they must remain too soft to be long worked on the board. The bread we have already baked is the kind preferred by most people, being light, spongy, and sweet. For eating when fresh, for making into milk toast, bread puddings, or griddle-cakes, and for crumbs with which to scallop or fry oysters, perhaps it is better for the spongy quality; but to a lover of bread and butter, pure and simple, bread made in the same manner, without potato, is best. It is light, but firmer and more compact in texture, and has a crisp sweetness excelling the other. In dry toast it is far better, having none of that husky emptiness which is a characteristic of dry toast made from spongy bread. Good bread may be made without potato, with very little labor. Beating it for a moment whenever flour is added, it need receive no other attention until stiff enough to be worked with the hand, when all the flour required can be worked in, during fifteen minutes' kneading, and the bread be placed immediately in the pans to rise. Made in this manner, and given only this small amount of time and labor, it will be found vastly superior to the bread at present disgracing a large proportion of American tables.

"Cousin Kate, why do you scald the flour with which you set the ferment for bread?"

"After trying various methods, I obtained the best results from so doing. Rising more slowly at first, the ferment appears to be quicker and more active, when its strength is most required to lighten the mass of flour. The scalding also prevents the ferment from souring; and it can, after becoming light, stand for hours without acquiring that yeasty smell and taste which spoils so much bread."

"Why do you use the wooden stick or spatula, instead of a spoon, for beating the sponge?"

"For several reasons. One is, that it is lighter; and the handle being round and smooth, does not fret the hand like metal. Then, it cuts through the dough more effectually than a spoon. My grandmother's hasty-pudding stick was the model after which this was made. It is a good thing to have several of these in a house. They should be made of various sizes, of hickory, ash, or other hard wood, and should be from a foot and a half to two feet long. The blade should be from one and a half to two and a half inches in width, and four or five in length, thickest in the middle, gradually growing thin toward the edges and square at the bottom. This stick is preferable to a spoon for beating cake or stirring mushes, and is quite indispensable in the fruit season, to keep small fruits stirred from the bottom of the preserving-kettle while cooking."

"Cousin Kate, all the cook-books that I have seen, agree that milk is better than water for making bread. Why do you not use it?"

"For the reason that I differ in opinion from those who think milk best. Milk that stands and ferments, usually acquires an unpleasant smell and taste. Sponge made with milk is more likely to sour; and bread made with milk, at the same time that it is less sweet in flavor, dries sooner, and does not keep so well. Lastly, water is cheaper, and within the reach of all.

"Soda, as used in many households is one of the greatest abominations of the kitchen. It is a malign intruder in all mixtures where an acid is wanting; and even where an acid is present, it should be used with great care and discretion. The introduction of baking-powders is a good thing; for, in these the acid and the alkali are more accurately proportioned than by careless cooks. Whenever I speak of a measure of baking-powder, I mean to designate the amount which the maker of the powder directs to be used in a given quantity of flour. Those baking-powders which are put up in separate parcels—the one containing the alkali, the other the acid—are perhaps preferable, but should only be used by those cooks who believe in careful and exact measurements. In some cases, tin measures accompany the powder; in others, a teaspoon is the measure designated. To attain exactness in these measurements, place a portion of the powder on the molding-board, and, with a knife, mash it smooth; then, fill the measure heaping full, and, with the knife, strike off all above the rim. Where exactness is indispensable, make even (never heaping) measures. Mix the quantity to be used with a small portion of flour by repeated siftings, which can be easily and quickly accomplished by the use of two pieces of strong soft paper. Place the sieve on one of these, and empty the flour from the other into it; shake through, and repeat it as often as desirable. In all batter-cakes or soft dough, where baking-powder is used, mix it in this manner with a small quantity of flour, which add near the close of the mixing process; but in biscuit and stiff dough, sift the baking-powder through all the flour used. Some cooks are affected with soda-mania. On general principles, as it seems, they put it in all breads and cakes. If not required to sweeten or make light, they confidently assert it will make tender. So the greens are plagued, the string-beans tormented, and the young peas cursed, by this evil spirit, the exorcism of which would be a blessing to thousands.

"For lunch to-day, Alice, you shall make some cream-biscuit which will take your mamma captive, and charm her as completely as any material substance can, if the biscuits you produce rival, as I hope they may, the puffy things made by my rosy-cheeked friend, Sally. Sally and I were girls together, and much of my summer rest was found at the old farm-house, where she and her sisters grew up, blossomed, and faded, as the years went by, in which I served my State as instructor of its youth. Through all my life, in which I am sorry to say hot biscuits have been a not infrequent event, I have never found those that quite equalled Sally's. She, however, was the only one in the family who invariably made them just right. If her mother or one of her sisters undertook the task, the cakes often came out of the oven yellow, and smelling of soda because of an overdose; or white and "sad," because too little had been used; for the exact amount required depends upon the acidity of the cream. Sally had the knack of guessing just right. Taking the pint bowl of sour cream in her hand, she would stir and taste it with a critical air; and giving a wise little nod, as if she had solved the riddle, proceed to measure a teaspoon half-full, even full, or heaping full, of soda, which she placed in a cup, and poured on it a spoonful of boiling water. Letting this stand, she made room in the middle of the flour for the cream; and, with her fingers, mixed the flour nearest the cream lightly in, adding a pinch of salt. While it was still a soft batter, she drained into it the soda, leaving any sediment there might be in the cup. Then her hands danced here and there through the dough, mixing it rapidly until stiff enough to roll out on the bread-board, to cut into round cakes, and place immediately in a quick oven.

In twenty minutes these cakes came out of the oven four times as thick as they went in, the most delicate puffy things imaginable; but *your* cream-biscuit, Alice, must be made with sweet milk and a little butter, instead of cream.

"To a quart of flour, add a measure of baking-powder, sifting the whole three or four times; a salt-spoon of salt, and two ounces of butter, so carefully rubbed through the flour that you can not find a particle the size of a pin-head. Add cold sweet milk, to make a soft dough; roll out, cut into cakes, and bake in a quick oven."

At lunch, Cousin Emmeline praised the biscuits, as she ate them with strawberries, which had been sprinkled with sugar, and set in the refrigerator for half an hour. The sugar, with its sweet art, had coaxed enough juice from the berries to dissolve itself; and, wrapped in this delicious syrup, the berries were far more palatable than when eaten sharply acid, with a portion of dry sugar. Alice was pleased with the praise her mother bestowed upon the biscuit, until it occurred to her that even a higher achievement had been possible. Then she asked, half reproachfully,—

"Cousin Kate, why did you not have me make a strawberry shortcake,—or don't you know how?"

"Oh, yes. You would have made the shortcake just like these biscuits; rolling the dough quite thin, not much thicker than pie-crust, and baking on pie-pans, the size of the pan. When baked, with a fork and your fingers separate the upper and under crust of the cake, spread the inside with butter, and, laying one half of the cake on a plate, cover it thickly with berries well sprinkled with sugar. Then add another half cake and another layer of berries, till the pie is thick enough, finishing with fruit. If you desire to disguise, and, in a measure, destroy the flavor of the berry, serve strawberry shortcake with sweet cream poured over it. The cream also renders it more indigestible. My opinion is that any one, after once eating that sweet, crisp loaf-bread, a day old, together with strawberries served like these, will never voluntarily run the risk of dispepsia, for the sake of hot biscuit or strawberry shortcake."

"Belonging to the bread-family, and a very genuine, respectable member thereof, is our round, chubby little travelling companion, known as 'Maryland Biscuit.' Although rarely met with outside the State whose name it bears, it is a very desirable acquaintance for travellers who provide their own lunches; for pic-nic parties, and for ladies who desire always to have the pantry well stored with good things. The Maryland biscuit occupies that happy medium state between a cracker and an ordinary biscuit or roll, and is by many preferred to either. To bring these cakes out of the oven perfect specimens of their kind, requires some labor, as it is necessary not only to knead, but also to pound or beat them with a heavy hammer or mallet. To four pints of flour add three ounces of lard, a teaspoonful of salt, and a pint of cold water. Rub the lard and salt through the flour until perfectly mixed, before adding the water. This proportion of water may not be the exact quantity in all cases, as some flour requires more, some less; but the dough must be very stiff, and rendered soft and pliable by working and pounding. When light, and well worked, the dough will 'blister,' and the pulling off of a small piece quickly will cause a sharp, snapping sound. When these signs appear, rest from the labor of pounding; gather between your fingers a little wad of the dough, pull it suddenly from the main lump, and mould it into a round mass, and lay it aside, face downward. In like manner fashion enough to fill the bread-pan. Then, one by one, pick up these round balls, and hold each for an instant in the hollow of your left hand. While nestled there so cozily, place your right thumb squarely in its face, enlarging equally its surface, and leaving a hollow in the center; place in the bread-pan, not allowing the biscuits to touch each other. Bake at once in a hot oven until done, and of a light-brown color. If the cakes are "sad," or heavy inside, when cold, they are not sufficiently baked, as they should be light, and of a fine even grain. When most perfect, these biscuits crack at the edges or sides, the upper and under crust being forced apart, in order to give a hint of the lightness and whiteness within. When your oven is sufficiently hot, twenty minutes is about the average time required to bake them properly.

"No Maryland supper-table is in company-order without these modest, unpretending little fellows, who are quite at home with fried oysters, and fit company for cold turkey and broiled chicken."

One morning at breakfast, Alice asked, "When may I be Queen of the Kitchen for a whole week, and do all the cooking without any supervision, Cousin Kate? I shall not feel really certain that I know how to cook so long as you have to oversee me. Besides, when are you to write that lecture on 'The Proper Rearing and Training of Children,' if you devote all your time to me and the kitchen?"

After reflecting a moment, I replied, "Your suggestion is a good one, Alice, and this plan occurs to me: For one week I will do the cooking, and you shall observe and help as heretofore; and, during the week, you shall note the bill of fare at each meal, and make such memoranda as you think necessary. Then, the week following, you may repeat or duplicate my week of cooking."

"I like that proposition," said Alice; and Emmeline suggested, "I hope you'll make a judicious selection, Kate, if we are to repeat the bill of fare,—good, bad, or indifferent."

The next day I began on the preparatory week, and on the evening of the seventh day thereafter, I abdicated in favor of Alice, who brought her notebook, and, while finding the place, murmured, "For breakfast one week ago to-morrow morning, we had —Oh, here it is!—Oatmeal mush, picked up codfish cooked with cream, baked potatoes, French twist reheated, coffee. (*Mem.*—At seven o'clock, when I entered the kitchen, Cousin Kate stood by the long table, knife in hand, bending over a codfish. She stripped off large pieces of the fish, then cut them across the grain, into pieces an inch long. As these were two or three inches wide, she picked them into small bits, throwing out the bones, and letting the fish fall into a pan of cold water. In a few minutes she had thus prepared enough for breakfast. Stirring it about in the water, she left it to freshen, while she prepared the oatmeal mush. Into a tin-lined stew-pan she put two pints of water and half a teaspoon of salt. When the water boiled, she sprinkled in slowly half a pint of oatmeal, stirring it with the mush-stick. The water was boiling quite rapidly. In two minutes the meal was all in, but she continued to stir, letting it boil for two or three minutes longer, when it appeared about the consistency of thick gruel or thin batter. Then she placed the stew-pan back on the range, where the heat was only enough to keep it simmering. Removing the stick and covering closely, she left it undisturbed till breakfast. Next, she took six potatoes of medium size, and, washing them very clean, placed them in a pan ready for the oven. Then she ground the coffee and put it to steep, not boil. She allowed a tablespoonful of coffee for each cup; and, placing it in the pot, added a cup of cold water, and set it back on the range to steep. If it chanced to boil, she added more cold water. Just before serving breakfast, she added as much boiling water as was needed, and allowed it to boil for a minute; then she lifted the pot to the kitchen table, and with a spoon removed the grounds which adhered to the inside of the pot, near the top. Rinsing these off, she poured out part of a cup, which she put immediately back, added a spoonful of cold water, and sent the coffee to the breakfast-table. When mamma poured it, three minutes later, it was clear as amber. Cousin Kate selected a bowl-shaped iron kettle holding about two quarts, and not a shallow pan or spider, in which to cook the codfish. She stirred the fish about in the water, and drained it in a tin strainer, then placed it in the kettle with half a pint of fresh water, covered it close, and let it boil for ten minutes, or until the water was all evaporated, and the fish well cooked. At this stage she added a pint of thin cream; and, when it boiled, two spoonfuls of flour stirred to a smooth paste in a little cold milk; let it boil for a minute or two, peppered it lightly, and served it. About ten minutes before breakfast she put the twist into the oven; and, from time to time, looked at it to be sure it was not burning. When the potatoes were baked, breakfast was served immediately. Cousin Kate lifted the pan containing them to the kitchen table; then, one by one, she picked up the potatoes in a cloth, which she held to protect her hand, gave each a gentle squeeze, until it opened its mouth, whence issued a puff of hot air; then placing them in a dish, sent them to the table.

When Alice had finished reading these notes from her memorandum-book, I said, "Correct, to the minutest particular." She smilingly answered, as she bade me good-night, "Then, let not your heart be troubled about breakfast, nor have a thought of care or responsibility. I shall ring the dressing-bell at half-past seven, and woe betide you and mamma, if you spoil my first breakfast by your tardiness."

Cousin Emmeline and I were on time next morning, and presented our Queen of the Kitchen with a bouquet of freshly-gathered flowers, in the arrangement of which we had spent some anxious moments. As I served the codfish, white, creamy, and tempting, I said, "Now *this* fills the spoon in a plump and satisfactory manner. I am very fond of codfish when properly cooked; and I find it terribly trying, sometimes, to have so appetizing a dish fraudulently represented by bits of fish half cooked, and salt as brine, straying forlornly about in a sloppy gravy. But this is just right; neither too thick nor too thin. In this artistic combination, it is difficult to decide where the fish ends and where the gravy begins."

Here Emmeline interrupted my rhapsody over the fish, by asking, "Where is the boiled milk for Kate's coffee, Alice?"

"There! I've forgotten the milk, Cousin Kate! But how did it happen that it was not down in my memoranda?"

"For the reason, I dare say, that boiling milk in a milk-boiler requires very little attention. You fill the inner boiler with milk and the outer one with water, and set it back on the range, where it simmers and sings to itself, until needed; and I suppose you neglected to make note of so trifling a matter."

"I am very sorry, indeed, Cousin Kate; for, to weaken your coffee with water, will spoil it."

"Not at all, when I have cream like this. But isn't it strange, Emmeline, that so indispensable a thing as a milk-boiler should be so rarely found in the kitchen?—and that the mistress of the house should go on, year after year, boiling milk in all sorts of pots and pans,—often scorching it so that it is unfit for use, wasting the milk, and spoiling her temper, all for the want of a milk-boiler which costs a dollar?"

"But supposing, Kate, she hasn't got the dollar, and if she asks her husband for it, he answers with contempt, 'Milk-boiler, indeed! My *mother* was one of the best cooks in the world, and she never heard of a milk-boiler!' What would you do in such a crisis, Kate?" "I don't know; but I have serious thoughts now, of writing a lecture, as soon as the one about children is finished, entitled, 'The Abuse of Power, or the Cause and Cure of Domestic Infelicity.' Here Alice laughed outright, saying, "Excuse me, Cousin, but it seems so funny that you who never had husband or child, should write upon these subjects."

"For that very reason, Alice, may I not be an important and unprejudiced observer? Commend me to an old maid or an old bachelor for true wisdom on these subjects."

Thus, in idle talk we whiled away an hour over Alice's first breakfast, which Emmeline and I pronounced a success, notwithstanding the forgotten milk.

---

Earn your own bread, and see how sweet it will be! Work, and see how well you will be! Work, and see how cheerful you will be! Work, and see how independent you will be! Work, and see how happy your family will be! Work, and before you know where you are, instead of repining, you will find yourself offering up thanks for all the numerous blessings you enjoy.

---

A great deal of water may be got from a small pipe, if the bucket is always there to catch it.

